# Antimicrobial activity of various ethanolic plant extracts against pathogenic multi drug resistant Candida spp.

**DOI:** 10.6026/97320630013067

**Published:** 2017-03-31

**Authors:** Shaista Khan, Mohd Imran, Mohammed Imran, Nuzhat Pindari

**Affiliations:** 1 Department of Biosciences, Integral University, Lucknow U.P. India;

**Keywords:** Candida, MDR, Plant extract, Antifungal activity

## Abstract

A total of 50 Candida isolates were isolated and identified from clinical specimens and these were tested for resistance to various antifungal drugs. It was observed multi-drug resistance in all candida isolates by 84%, 62%, 60%, 76%, 46, 30%, and 22% against fluconazole, clotrimazole, Amphotericin B, itraconazole, ketoconazole, miconazole and nystatin tested respectively. The isolates, which were found to be resistant to antifungal drugs were selected and subjected to antifungal testing against six ethanolic plants, extract namely Azadiracta indica, Allium sativum, Cordia dichotoma Ocimum sanctum, Syzygium cumini and Trigonella foenum grecum. All the plant extracts tested were found to effective against all MDR Candida isolates with inhibition zone ranging from 10- 18mm in diameter. Ethanolic extract of Allium sativum was observed most effective against the isolates among all the plants extracts tested. The minimum inhibitory concentration (MIC) of all ethanolic plant extract was recorded ranging from 1.56-25mg/ml against MDR candida isolates. Phytochemical analysis of the alcoholic plant extracts revealed the presence of alkaloid, flavanoid, glycosoid, phenol; phenol, tannins, saponins in all the plants studied. The present study may be successful in identifying the plants with different antimicrobial activity. These plants containing various phytochemicals may be exploited in the treatment of infectious diseases caused by drug-resistant microorganisms.

## Background

Antibiotics provide the main basis for the therapy of microbial infections. However, overuse of antibiotics has become the major factor for the emergence and dissemination of multi-drug resistant strains of several groups of microorganisms [1]. Candida species have become the leading pathogens responsible for nosocomial bloodstream infections with C. albicans causing more than 50% of these infections [2]. Candida species are now recognized as major agent of hospital-acquired infection [3]. Candidiasis is caused by different species of fungi belonging to the genus Candida especially C. albicans. It is found mainly as secondary infection in individuals with some underlying immunocompromised condition and very rarely as the primary disease [4]. More recently, azole antifungal compounds, with lower cytotoxicity and perfect efficacies, have emerged as the main drugs used in treatment of Candidal infections [5]. However, prolonged use of azoles has led to the development of drug resistance in C. albicans and other species. Non albicans Candida like C. tropicalis, C. krusei, C. glabarata and C. parapsilosis are less susceptible to azoles, particularly fluconazole [6]. Fluconazole and Amphotericin B are generally used against human pathogenic fungi but these show some side effects and toxicity. The slow pace of newer antibiotic development coupled with the availability of fewer antifungal agents with fungicidal actions centered on inhibition of ergosterol synthesis has provided the need to discover nature in search of herbal medicines with novel targets and mode of actions [7]. Researchers are trying to develop better herbal products against MDR pathogens due to the short active life of newly made antimicrobial drugs.

In the present scenario of emergence of multiple drug resistance to various pathogenic candida species, this has necessitated a search for new antimicrobial substances from naural sources speacially medicinal plants. In recent years, antimicrobial properties of medicinal plants are being increasingly reported from different parts of the world [8-16]. The selection of medicinal plants is based on their traditional uses (06 plants) in India [17-19]. The aim of this study is to design new natural therapeutic ways against multi drug resistant Candida species.

## Methodology

### Sample Collection

A total of 109 clinical specimens consisting of pus swab, sputum, urine, gastric aspirate and blood samples were collected from the central pathology laboratory of Integral Institute of Medical Sciences and Research Lucknow in a sterile container (containing stuart’s transport medium) and stored at 4°C for further processing.

### Isolation and identification of the isolates

The specimens collected were directly streaked onto HiCrome Candida Differential Agar. The plates were incubated at 37°C for 24-48 hours. Smooth colonies of different colors Candida albicanslight green, Candida tropicalis- blue, Candida krusei- purple fuzzy, Candida glabrata-cream) were observed. These colony were further confirmed by Germ tube test that shows the ability of the isolates to germinate inside a tube, was performed for all the 50 samples. Following three hours of incubation in serum at 37°C, samples were examined under the microscope for their ability to germinate. The isolates were streaked on Sabouraud Dextrose Agar (SDA) and store at 4° until use [6].

### Collection and authentication of plant materials

Healthy leaves of Ocimum sanctum, Azadirachta indica, Syzygium cumini and Cordia dichotoma, were collected from the Herbal Garden of Faculty of Pharmacy, Integral University, Lucknow and roadside of Kursi road, Lucknow. Bulbs of Allium sativum and seeds of Trigonella foenum- graecum were purchased from local market. The plant materials were washed; shade dried and powdered by hand crushing. All plants were authenticated by Department of pharmacy, Integral University Lucknow.

### Preparation of plant extracts

Hundred (100) g of dry powder of plant material was soaked in 100ml of ethanol for 5 days with intermittent shaking and at the end of extraction, the extract were filtered through Whatman filter paper No. 1 (Whatman Ltd., England) to make a crude ethanol extract. The filtered extract was left to dryness under reduced pressure on rotary evaporator at 40°c and stored at 4°c for further use [20]. Stock solution of crude extracts at different concentration (50 mg/ml, 100 mg/ml, 200 mg/ml, and 300mg/ml) was prepared for antimicrobial assay.

### Phytochemical screening

The extracts of the dry powdered leaves and seeds analyzed for the presence of various phytoconstituents like flavanoid, alkaloid, glycosoid, phenol, tannin and saponin [21].

### Determination of antimicrobial resistance

Pure isolates of identified Candida spp. were subjected to antimicrobial susceptibility testing using the disc diffusion method as recommended by Kirby Bauer method according to the recommendations of Clinical Laboratory Standard Institute [22], using the following antifungals discs Amphotricin B, Clotrimazole, Fluconazole, Itraconazoel, Ketoconazole, Miconazole, Nystatin obtained from Hi-Media Laboratories, India. The presence of a clear zone around the antibiotic disc is measured with meter rule in millimeter (mm).

### Antimicrobial activity of plant extracts

Antimicrobial activity of plant extract was carried out by agar well diffusion method [21]. 15 MDR isolates of Candida spp were selected for antimicrobial screening. 0.1 ml of diluted inoculums (105 CFU: ml) was spread on the SDA; wells were made on the medium by using 6 mm cork borer. The dried plant extracts were dissolved in dimethyl sulfoxide (DMSO) to make final extract concentration 300, 200, 100, 50, mg⁄ml. Each well was filled with 50 μl of plant extract, incubated at 37°C for 24 h. Zone of inhibition around of each well was measured in millimeter. DMSO and ethanol was used as a negative control and an antibiotic from which the isolates were sensitive was used as positive control.

### Determination of MIC of plant extracts

To determine MIC of plant extracts the broth micro-dilution method was performed [23] with some modifications. The inoculums of the tested isolates were prepared using the colony suspension method. Ninety-six-well culture plates were used, and serial two-fold dilutions of the extracts were dispensed into the plate wells. Two-fold dilutions of nystatin were used well along with 150μl of Mueller Hinton Broth. 30μl of broth culture was added to the wells. Three control wells were maintained for each test batch; the positive control (antibiotic, Mueller-Hinton broth and test organism) and sterility control (Mueller-Hinton broth and DMSO) and negative control (Mueller-Hinton broth, test organism and DMSO). The plates were incubated at 37 °C for 24 h. The fungal activity in the test wells was detected by adding 40 μL of 0.2 mg/ml of 2-(4-Iodo phenyl)-3-(4-nitro phenyl) 5- phenyltetrazolium chloride (I.N.T.) (Himedia, India) solution dissolved in sterile distilled water to each well. The plates were incubated for further 30 min, and estimated visually for any change in color to pink indicating reduction of the dye due to bacterial growth. The lowest concentration (highest dilution) of the plant extract required to inhibit visible growth of the tested microorganism was designated as the MIC.

## Result

**Figure 1:**
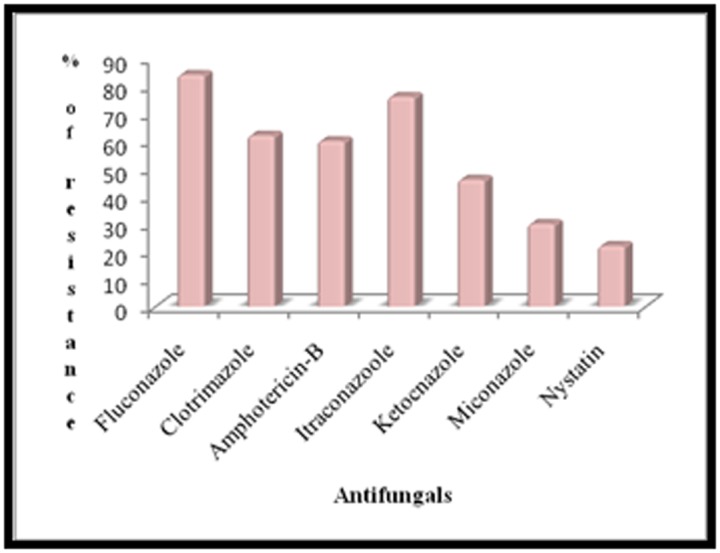
Percentage of antifungal resistance in Candida isolates

51 bacterial samples were taken for the study, one of which was MTCC strains and fifty were of clinical origin. The latter were tested as per CLSI guidelines and found to be multi-drug resistant with several antifungals. The results given in (Figure 1) showed Candida albicans strain resistance to 84% of fluconazole followed by 76%, 62%, 60% and 46% to itraconazole, clotrimazole, amphotericin-B and ketoconazole respectively. Candida albicans are less sensitive against nystatin and miconazole (22% and 30%). Qualitative phytochemical analysis was carried out for all of six the plants extract. Qualitative phytochemical analysis was carried out for all of the plants. Plants contained alkaloids, flavonoids, glycosides, phenol, tannin and saponin, which could be attributed to the significant antibacterial activities that were recorded. The results of phytochemical analysis of all these extracts were recorded (Table 2). In this study, all six plants, the crude extracts of Allium sativum, Azadiracta indica, Ocimum sanctum, Syzygium cumini, Trigonella foenum-graecum and Cordia dichotoma showed good antimicrobial activity against multidrug resistant isolates of candida isolated from clinical specimens. Allium sativum was found to give the most potent antimicrobial extract with maximum inhibition zone size which is 18mm in isolate C9 whereas Trigonella foenum-graecum showed minimum antifungal activity with inhibition zone size 8mm against C13 at concentration 300mg/ml. Azadiracta indica showed highest growth inhibition against isolates C2 and C10 at 300mg/ml (17mm) while maximum growth inhibition was observed in the case of C9 at 100 and 200 gm/ml of the extract tested. It was observed minimum inhibition growth in Candida at 50mg/ml of the extract. Ethanolic extract of Allium sativum sowed highest antifungal activity against C9 with growth inhibition zone 18 mm and 16mm at 300 mg/ml and 200 mg/ml respectively. Isolates C2, C3, C4 and C13 showed inhibition of their growth with 9 mm in diameter at 50-100 mg/ml against ethanolic extract of Allium sativum. Maximum growth inhibition of isolate C11 was recorded at 300 mg/ml of Ocimum sanctum extract. This extract showed maximum antifungal activity at 200 mg/ml against C9 and C10 isolates (14mm) whereas less activity was observed at 50 mg/ml against isolates C2 and C11. As observed in (Table 3), Syzygium cumini showed the maximum growth inhibition against isolate C10 (16 mm) at 300 mg/ml whereas at 200 mg/ml, 14mm growth inhibition was recorded against isolate C9. Less inhibition was observed at lower concentration (50 mg/ml). In Trigonella foenumgraecum maximum growth inhibition (15mm) was recorded against isolates C4, C9 and C11 at 300 mg/ml and similar results were observed against C2, C11 and C13 at 200 mg/ml. Cordia dichotoma also have a high antifungal activity against C2, C10 and C11 isolates at 300 mg/ml while at 50 mg/ml significant growth inhibition of isolates C1, C2 and C4 was also recorded. Isolate C9 was observed a most susceptible (17-18 mm) to all the six ethanolic extracts tested. MIC values of ethanolic extracts of six plants were evaluated. Azadiracta indica had 1.56 mg/ml as the lowest MIC value against C9 isolate and 0.78 MIC value against MTTC strain. MIC value of Allium sativum was 1.56 against C2 and C10 whereas Ocimum sanctum had also 1.56 MIC value against C11, C13. C9, C10 showed lowest 1.56 mg/ml MIC in Syzygium cumini whereas MTCC strain had MIC 6.25mg/ml in same extract. Trigonella foenum-graecum and Cordia dichotoma both showed same MIC value which was 1.56 mg/ml in MDR candida isolates. The MIC values as a result of all six-plant extract for all 15 MDR Candida isolates are presented in (Table 4).

## Discussion

Due to excessive use of antibiotics, increase in antimicrobial resistance has been observed among the microbes including bacteria and fungi. In our study of antifungal resistance to antifungal among the candida isolates was studied. It was observed multi-drug resistance in all candida isolates by 84%, 62%, 60%, 76%, 46%, 30%, and 22% against fluconazole, clotrimazole, Amphotericin B, itraconazole, ketoconazole, miconazole and nystatin tested respectively. Minority of the isolates also showed sensitivity against the all tested antibiotics. It was also found seven patterns of resistance in different combinations against candida isolates. Our findings are similar to the results obtained by the other workers. It was observed that clotrimazole resistance was present in C. albicans isolated from HIV-infected patient [24]. All tested Candida isolates were susceptible to nystatin, miconazole, ketoconazole and fluconazole and C. albicans isolates were more susceptible to azoles than was C. glabrata [25]. In addition, only 6% of C. albicans isolates were resistant to fluconazole. Previous study showed that 90.2% and 91.4% of isolates of Candida species were sensitive to fluconazole and ketoconazole, respectively [26]. Whereas, 85.1% and 76.1% of tested isolates were resistant to fluconazole and econazole, correspondingly and their study showed that 100% of nonalbicans candida species, were resistant to fluconazole [27]. Many studies were undertaken to find the development of resistance among Candida species such as 34.2% resistance to fluconazole [28]. While 9.9%, 8.4%, 4.3%, and 25% were also recorded [29-32].

The present study has been undertaken to determine the antimicrobial activity of plant extracts of six medicinal plants namely, Syzigium cumini, Azadirachta indica, Allium sativum, Cordia dichotoma, Trigonella foenum grecum and Ocimum sanctum in ethanolic solvent. The significance of our study is particularly important keeping in view the growing resistance of the microbial both bacterial and fungal species to commercially available antibiotics. Because of the emergence of many resistant strains against commonly used antibiotics, the researchers are trying to evaluate some medicinal plants as the alternative of antibiotics. The various kinds of plants have antibacterial and antifungal activity containing effective phytochemicals. The phytochemical analysis of the plants under study showed that the ethanolic extracts of Azadirachta indica, Syzigium cumini, Allium sativum, Cordia dichotoma, Trigonella foenum grecum and Ocimum sanctum had alkaloid, tannins, saponins, phenols, flavonoids and glycosoids. Our phytochemical analyses are in agreement with the reports of other workers [17, 33-36]]. These plant extracts need to be correlated to the antimicrobial activity. All the fifteen of Candida spp. namely, C. albicans, C. tropicalis, C. krusei, C. glabrata were resistant to the standard antifungal agents including fluconazole, itraconazole, miconazole, clotriconazole, ketoconazole, amphotericin-B and nystatin Interestingly, ethanolic extracts of these plants produced fairly larger zones of inhibition, particularly the extract of Allium sativum showed highest activity with zone of inhibition of 18mm against isolate C9. Extracts of Azadirachta indica, Cordia dichotoma, Syzigium cumini, Trigonella foenum grecum and Ocimum sanctum also showed a significant activity against C10 (17 mm), C11 (17 mm), C2 (17 mm), C10, C11 (17 mm) and C10 (16 mm) and C4, C9 and C11 (15 mm) respectively at 300-mg/ml concentration tested. This finding is valuable as the extracts could provide an alternative to fluconazole, itraconazole, miconazole, clotriconazole, ketoconazole, amphotericin –B and nystatin tested against Candida spp. Although the phytochemical analysis of these compound has been done but further studies are required to pin point the active compounds. Other workers have also observed higher zones of inhibition with ethanolic extract of Azadirachta and trigonella, which supports our observations [37,38]. Our findings are in agreement of other workers [39,40]. 14 mm inhibition zone were by Syzigium cumini against Candida albicans [41]. There are very close results were recorded with Trigonella foenum graecum against Candida species [42]. Tulsi ethanolic extract was found to be more active against C. albicans in comparison to A. niger [43]. Researchers were observed that ethanol extracts of Allium sativum (Garlic) and Syzigium cumini have antifungal activity on Candida albicans and 11.6mm zone of inhibition was recorded [44,12]. The Syzygium cumini leaves hydro alcoholic extract displayed the highest level of activity against Candida krusei. The inhibition zones varied between 8.3 ± 0.3 mm and 14.7 ± 0.3 mm. The antifungal activities of some spices and herbs also have been reported against Candida [45]. Cordia dichotoma leaves are very rich in saponins, and flavonoids. The presence and large amount of tannins also confirms its astringent property [46]. The saponin content makes the leaves an important source of detergents; surface-active agents used in industrial applications and also possess beneficial health effects [47] including antimicrobial characteristics. Our results revealed a strong activity of ethanolic solvent of Syzygium cumini, Azadirachta indica, Allium sativum, Cordia dichotoma, Trigonella foenum grecum and Ocimum sanctum with MIC varying from 1.56 to 25 mg/mL; 1.56- 25 mg/ml. our MIC results are also in agreement of the other workers [48-50].

## Conclusion

It can be concluded that the alcoholic extracts of different plant extracts have a significant activity against multi-drug resistant pathogenic candida spp. The obtained data are also comparable to the commonly used antifungal antibiotics such as fluconazole, itraconazole, clotrimazole, miconazole, ketoconazole and Amphotericin-B. These plant extracts may be the potential alternatives of antibiotics to avoid their overuse and side effects on human health and environment. Further studies are also required for evaluating their clinical efficacy.

## Figures and Tables

**Table 1:** Antifungal résistance pattern of 50 Candida isolates from clinical samples

No. of Antibiotics	Resistance Pattern	No. of Resistant Isolates	Percentage (%) n=50
1	IT	1	2
2	IT, FLU	4	8
	KT, IT	1	2
	AP, IT,	1	2
	FLU, NS	1	2
	FLU, CC	1	2
	CC, NS	1	2
3	FLU, KT, IT	3	6
	FLU, KT, CC	1	2
	FLU, IT, CC	1	2
	FLU, IT, NS	2	4
	FLU, IT, AP	4	8
4	IT, KT, FLU, MIC	1	2
	ITFLU, MC, CC	1	2
	FLU, IT, KT, CC	1	2
	KT, FLU, AP, CC	2	4
	FLU, IT, CC, NS	1	2
	FLU, AP, IT, NS	1	2
	FLU, APIT, MIC	1	2
	IT, FLU, AP, CC	1	2
	IT, KT, FLU, MIC, CC	2	4
5	FLU, IT, CC, MIC, NS	1	2
	FLU, KT, AP, MIC, CC	2	4
6	NS, CC, FLU, AP, KT, IT	1	2
	IT, FLU, KT, MIC, CC, AP	1	2
	FLU, CC, KT, MIC, AP, NS	1	2
	IT, FLU, CC, KT, MIC, AP	3	6
	FLU, MIC, AP, KT, CC, IT	1	2
	IT, FLU, CC, AP, KT, MIC	1	2
7	IT, KT, NS, FLU, AP, CC, MIC	1	2
Antifungal agents: IT= itraconazole, KT= ketoconazole, NS= nystatin, FLU= fluconazole, MIC= miconazole, CC= clotrimazole, AP= amphoterecin-B			

**Table 2:** Preliminary phytochemical screening of different ethanolic plant extracts.

Name of plants	Name of Phytochemical							
Parts used	Approx. Yield per 100 gm dry powder (mg).	A	F	G	P	T	S
Ocimum sanctum	Leaves	30	_	_	+	+	+	+
Azadirachta indica	Leaves	30	+	_	+	+	+	+
Allium sativum	Bulb	40	+	+	+	-	+	+
Syzgium cumini	Leaves	30	_	_	_	+	+	+
Trigonella foenum graecum	Seed	110	+	+	_	+	+	+
Cordia dichotoma	Leaves	45	_	_	+	+	+	-
Phytochemical key: A = Alkaloid; F = Flavanoid; G = Glycosoid; P = Phenol; T = Tannin; S = Saponin; (+) denote present; (-) denote absent.								

**Table 3:** Antifungal activity of alcoholic extract of six plants against MDR Candida isolates

Plant Extract	Diameter of zone of inhibition (mm)																MTCC
Conc. mg/ml	C1	C2	C3	C4	C5	C6	C7	C8	C9	C10	C11	C12	C13	C14	C15	
Azadiracta indica	50	-	8	7	-	-	-	-	-	8	-	-	-	-	-	-	-
	100	-	10	9	8	-	-	10	10	12	10	9	-	9	-	8	10
	200	9	14	13	12	8	8	14	13	15	13	13	9	13	-	8	13
	300	10	17	16	13	9	10	15	15	16	17	15	10	15	8	10	17
Allium sativum	50	-	-	-	-	-	-	-	-	8	9	-	-	9	-	-	0
	100	8	9	9	-	9	-	-	8	10	11	11	-	9	-	-	14
	200	11	11	14	9	10	-	9	13	16	14	15	8	12	7	9	15
	300	13	17	15	14	11	-	11	16	18	17	17	11	14	9	9	19
Ocimum sanctum	50	-	8	-	-	-	-	-	-	-	-	8	-	-	-	-	8
	100	9	11	-	9	-	-	9	8	10	9	12	-	10	-	-	10
	200	10	11	9	10	-	-	9	12	14	14	13	-	11	-	9	13
	300	14	14	10	11	-	9	10	13	15	15	17	10	16	-	12	15
Syzygium cumini	50	-	-	-	-	-	-	-	-	-	-	7	-	8	-	-	-
	100	8	9	8	8	-	-	9	10	10	9	10	8	10	-	8	-
	200	9	12	10	11	7	9	9	12	14	13	13	11	12	-	12	10
	300	13	14	13	14	10	11	13	12	15	16	14	12	13	10	14	13
Trigonella foenum-graecum	50	-	9	-	-	-	-	-	-	8	-	-	-	7	-	-	-
	100	-	9	-	9	-	-	8	9	12	10	12	-	11	-	9	9
	200	10	15	-	11	9	-	9	11	15	13	13	9	15	-	10	11
	300	11	13	10	15	11	-	13	14	15	14	15	10	13	8	13	14
Cordia dichotoma	50	8	8	-	8	-	-	-	-	-	-	-	-	-	-	-	-
	100	11	11	-	8	-	-	8	10	9	14	10	-	8	9	-	9
	200	12	16	9	11	9	7	10	12	15	17	16	-	10	11	9	15
	300	15	17	10	15	9	9	13	14	16	17	17	9	13	14	10	18
C1-C15; Candida isolates, MTCC; standard strain of Candida albicans																	

**Table 4:** MIC of ethanolic plant extracts against MDR Candida isolates

Candida isolates	MIC(mg/ml)					
A. indica	A. sativum	C. dichotoma	O. sanctum	S. cumini	T. feonum- grecum
C1	25	-	6.25	6.25	12.5	12.5
C2	6.25	1.56	3.12	12.5	3.12	6.25
C3	12.5	3.12	-	-	6.25	-
C4	12.5	25	6.25	25	6.25	3.12
C5	25	-	-	-	-	12.5
C6	-	-	-	-	25	-
C7	25	12.5	6.25	-	6.25	12.5
C8	6.25	6.25	12.5	25	25	3.12
C9	1.56	3.12	3.12	3.12	1.56	1.56
C10	3.12	1.56	3.12	3.12	1.56	6.25
C11	3.13	12.5	1.56	1.56	3.12	1.56
C12	25	-	-	-	25	-
C13	12.5	6.25	12.5	1.56	25	3.12
C14	-	-	6.25	-	-	-
C15	-	-	-	12.5	6.25	6.25
MTCC	0.78	0.78	3.12	1.56	3.12	3.12
MIC: minimum inhibitory concentration, C1-C15: Candida isolates tested, – : No activity at the concentration of the extracts tested.
